# Epidemiological criteria for causation applied to human health harms from RF-EMF exposure: Bradford Hill revisited

**DOI:** 10.3389/fpubh.2025.1559868

**Published:** 2025-05-27

**Authors:** John William Frank

**Affiliations:** Usher Institute, University of Edinburgh, Edinburgh, Scotland, United Kingdom

**Keywords:** radiofrequency electromagnetic fields (RF-EMF), cancer, epidemiology, causation, narrative review

## Abstract

**Purpose:**

This paper reviews the applicability of standard epidemiological criteria for causation, to the multidisciplinary studies of RF-EMF exposure and various adverse biological and health effects, with the aim of demonstrating that these criteria, although 60 years old, are still helpful in this context—albeit in some cases not entirely straightforward to apply.

**Methods:**

This is a commentary, based on Bradford Hill’s criteria for assessing evidence of causation, applied to recent primary studies and systematic reviews of the RF-EMF/health-effects literature. Every effort has been made to use non-epidemiological language to reach a wide readership of biologists, physicists, and engineers now active in this field.

**Results:**

A rapidly growing number of human observational epidemiological studies have assessed the association of diverse adverse health effects with RF-EMF exposures. However, existing systematic reviews and meta-analyses of these primary studies have substantially diverged in their conclusions. The application of Bradford Hill’s epidemiological criteria for assessing evidence of causation, originally designed for use in occupational and environmental health, casts light on some of reasons for this divergence, mostly reflecting the key weaknesses in the primary literature, which are discussed in detail. As a result of these threats to their validity—particularly the facts that (1) exposure measurement is typically subject to substantial error, and (2) insufficient time has elapsed, since modern cell phone use began in earnest, to allow tumors of longer latency to develop—most primary studies to date, and therefore many published systematic reviews of them, probably underestimate the true potential for causation, if in fact this association is causal.

**Conclusion and recommendations:**

In view of these findings, international experts representing professional and scientific organizations in this field should convene an independent Guidelines development process to inform future epidemiological studies of associations between RF-EMF exposures and human health outcomes. Wide dissemination of such Guidelines could help researchers, journals and their reviewers in this field to execute, review and publish higher-quality studies to better inform evidence-based policy.

## Background

Considerable scientific disagreement has developed in recent years over whether or not existing exposure limits and associated health and safety regulations in most Western countries adequately protect the public from potential health risks putatively associated with radio-frequency electromagnetic fields (RF-EMFs) (1–16). Indeed, over the last few years, a set of about 12 Systematic Reviews (SRs) of these various health effects, commissioned by WHO (17, 18) has led to substantial criticisms in the peer-reviewed literature (19–22), with extensive rebuttals by the Reviews’ authors (23–25) followed by retorts from the critics (8). Central to this controversy is well-established epidemiological guidance for assessing the extent to which observational (typically cohort and case–control) studies of RF-EMF exposure and various health outcomes, as well as systematic reviews of those studies, convincingly demonstrate features of such exposure-disease associations which are typical of causation.

The most widely used set of such causation criteria are those created by the eminent British statistician Austin Bradford Hill in 1965 (26). These are summarized in Table 1, using modern phrasing that reflects more recent thinking on their application to environmental epidemiological studies of ambient hazards, which have particular challenges to their design and analysis (7, 27–31). One publication to date has attempted a similar analysis (32) but is now a dozen years old, since which time the relevant literature has mushroomed. In particular, the entire field of RF-EMF health-effects is fraught with major disagreements about which specific measures of exposure—including cell-phone use proxies for such exposures—are most relevant, as well as feasible to implement in human epidemiological studies so as to facilitate high levels of consistency across study findings, enabling the literature’s capacity to demonstrate replicability (33, 34).

**Table 1:** Bradford Hill (26) criteria for assessing evidence of causation vs. association human health [This table is based on previous related materials (7, 39, 40)].

1. What is the observed **strength of the association** for the specific research question addressed, as measured by Relative Risk (RR) in cohort studies or Odds Ratio (OR) in case control studies?
2. Is the **strength of association** consistent across the studies reviewed? [This implicitly includes “Has the **quality of the primary studies** been robustly and objectively assessed?”]
3. Was there evidence of “**specificity**” of the putative health effect, to the particular exposure studied?
4. Temporality: Was the **timing of the exposure in question clearly prior to the onset of the health outcome** it is being linked to?
5. Is there a **dose–response relationship** between various levels of exposure to the putative cause of the outcome, and the magnitude of the Relative Risk for that outcome?
6. Has the **biological plausibility** of (i.e., credible mechanisms for) the putative causal relationship been established?
7. Has **“coherence”** of the putative causal association been demonstrated?—i.e., if the association were causal, would that be consistent with the known spatial and temporal distributions (i.e. the descriptive epidemiology) of the exposure and the outcome, over different populations over time?
8. Is there **experimental evidence of the reversibility** of the putative causal association?
9. Is there a clear **analogy** between this putative causal relationship and another, proven causal relationship in humans or animals, involving a similar sort of exposure and a similar sort of outcome?

## Methods

This paper reviews the relevance of each of the above Bradford Hill criteria for causation to the literature on RF-EMF and human health effects, describing the results of applying each criterion to the primary and secondary (review) literature in this field. A particular effort has been made to explain these findings in plain language suitable for non-epidemiologists, since the field of RF-EMF safety is interdisciplinary.

## Results

### Strength of association

Epidemiological studies have traditionally quantified the association between an exposure and any dichotomous health outcome (e.g., a disease) as a Relative Risk (RR) in *cohort studies* that follow large numbers of exposed and unexposed persons to determine what proportion of them develop the disease in question (29, 31, 35, 36). Virtually unique to epidemiology, an alternative study design does the reverse, taking a representative sample of patients with the disease in question, as well as controls without the disease (but at risk of it in future) and analyzing the proportion of each group which has been exposed in the past to the putative hazard under investigation. In such “*case control*” studies, an estimator of RR—the odds ratio—closely approximates the RR that would be found in cohort when the outcome is relatively rare, or the study is designed to ensure that the controls are questioned about their past exposure at the same calendar times as the incident cases occur (31).

Epidemiologists over several decades have developed an informal consensus about how large a relative risk needs to be for it to be solidly suggestive of causation—well over 2, and ideally over 3 or even 4 (31, 37). This is because common weaknesses in cohort and case–control study design and analysis can readily lead to biased RR estimates, and mis-state the strength of small relative risks typical of weak associations (31). Indeed, some decades ago one of the fathers of modern epidemiology, Sir Richard Doll, wrote a critique of unsound preventive advice based primarily on “weak effects” in observational epidemiological studies (38). Subsequently, even stronger critiques have appeared of conclusions about causation or prevention which are derived solely from studies with weak associations (37, 39, 40).

Environmental epidemiologists, however, understand that even small relative risks between 1.1 and 1.5, if unbiased, can lead to large population burdens of disease attributable to the hazard in question, if that hazard is widespread in a susceptible population (27, 29). This is easily demonstrated by the calculation of Levin’s “population attributable fraction,” (41) which makes use of both the RR indicating the strength of association of a causal relationship, and the prevalence of any given risk factor with that RR, in a specific population (29). For example, if a putative causal exposure has only a weak RR linking it to an adverse health outcome, such as 1.5, but that exposure affects, say 50% of the population (as, for example serious air pollution does in some global settings) then the proportion of the burden of cases in that whole population attributable to the exposure would be 33%—hardly trivial. Environmental epidemiological studies of widespread exposures (such as RF-EMF nowadays) must therefore walk a narrow path between maintaining vigilance against bias, while at the same time being open to finding small RRs of potentially great public health importance.

In the case of RF-EMF exposures and the major health outcomes that have been most frequently studied to date—such as brain tumors, a particularly large and longstanding literature—a quick glance at recently published systematic reviews (34, 42, 43) reveals many pooled RR estimates across primary studies of less than 2, with only a handful of sub-analyses of longer-latency associations (i.e., evident only after more than a decade has passed since exposure began, and so apparent only in studies published more recently) approaching, and occasionally exceeding, 3. Indeed, the highest RR reported by Moon et al. (34), is 3.53 from the Interphone study of acoustic neuroma and habitual past patient use of cell phones ipsilateral to (on the same side of the head as) the tumor [We shall return to this finding below, under “dose–response relationships” because it is arguably an example of evidence supporting such a relationship.]. On the other hand, critics of this study have run simulations which indicate that this excess risk in the heaviest user group is likely the result of a combination of systematic and random errors (44).

### Consistency of strength of association (across primary studies)

The standard epidemiological approach to assessing whether primary studies of a given association provide consistent—and therefore credible, in the sense that all sound science should be replicable—estimates of strength of association (RR/OR) of that association, is the systematic review (SR). SRs may or not be accompanied by meta-analytic pooling of the various primary studies’ estimates of RR/OR, according to an explicit judgment made by the review authors, guided by well-developed and now-quite-sophisticated *substantive and statistical* criteria for when such pooling is appropriate, based on formal assessment of the *heterogeneity* of both designs and analyses, as well as RR/OR findings, across the primary studies.

As may be obvious, however, one cannot proceed to that stage of a systematic review without first: (a) conducting a thorough and well-documented literature search (to allow later replication by others) for all the studies of relevance—ideally published and unpublished (45); (b) applying standard, detailed criteria to assess the *quality* of the primary studies, so as to eliminate those found to have design or analytic flaws likely to have led to significantly biased findings—an issue is discussed in the section immediately below. Only then can a valid assessment be made of whether the final short-list of high-quality primary studies of a given exposure-outcome association demonstrates sufficiently *homogeneous* findings to warrant their being pooled with meta-analytic statistical techniques (31, 36, 45).

#### Assessing the quality of the primary studies

In the past decade, numerous methodological tools have been developed for assessing the quality of observational epidemiological studies of putative environmental health hazards, as well as for guiding the rest of the process of synthesizing these studies in systematic reviews/meta-analyses: (1) the NIH OHAT “Risk of Bias” tool (46), and the associated “Navigation Guide” (47); (2) a shorter set of 10 “critical appraisal questions” specifically designed for biologists unfamiliar with epidemiology (48); (3) the COSTER framework for systematic reviews in environmental health (49, 50); (4) the international PRISMA guidance for systematic reviews (51); and (5) the relatively short and simple-to-use Oxford Center for Evidence-Based Medicine Critical Appraisal Questions for Systematic Reviews of Observational Studies of Causation (45). Notably, at least one study has shown that applying these tools yields quite different results (52).

Each of these alternative approaches to the quality assessment of both primary studies and SRs in environmental health, presents significant challenges even for an experienced epidemiologist. The OHAT tool is overly complex, with considerable internal duplication—as well as much jumping back and forth between experimental and observational studies. Yet non-experimental, observational cohort or case control studies, are pretty much all we have for serious human health effects, such as neoplasms, of RF-EMFs, due to the ethical constraints on experimental designs for putatively hazardous exposures. In addition, as noted in a recent critique (53), the OHAT risk-of bias tool does not cover some important aspects of SR methodology of major relevance to observational epidemiological studies—especially the need to carefully analyze the designs and analyses of the relevant primary studies for substantive *heterogeneity in design, analysis and inference*, in addition to conducting formal statistical tests for heterogeneity across the results of those studies (covered in detail below). The PRISMA guidance is helpful but designed primarily for meta-analysis of clinical epidemiological studies of medical treatments, where experimental study designs reign supreme, rather than primarily observational studies of widespread ambient environmental exposures such as RF-EMFs, where randomized controlled trials are typically unethical or impractical.

Finally, many of the recently published SRs of diverse biological and human health effects utilize the GRADE approach to assessing the confidence with which the primary literature on a given health outcome and RF-EMF exposure can be said to show convincing evidence of a consistent association, compatible with causation. However, the GRADE approach was also specifically designed for assessing evidence of medical-treatment efficacy across randomized controlled trials (54) rather than the observational epidemiological studies which almost exclusively form the body of evidence for biological and health effects of RF-EMFs. It is thus not surprising that the GRADE approach systematically discounts the value of non-randomized studies—thereby rendering it less than ideal for assessing the strength of evidence for fields such as the environmental epidemiology of serious health effects such as neoplasms, where RCTs are hardly ever feasible. A promising improvement to the GRADE approach is the well-established STROBE guidance [Strengthening Reporting of Observational Epidemiological Studies—(55)] for ensuring that key quality indicators are assessed across primary studies and in systematic reviews, in fields such as RF-EMF biological/health-effects where virtually all the evidence in human beings is non-experimental.

To reiterate, all of the various international guidelines for conducting, and reviewing the robustness of, SRs in environmental/toxicological epidemiology require, prior to any examination of across-study heterogeneity, a systematic screening of all the relevant primary studies for their quality. However, in the case of RF-EMF exposures and their putative health effects, all the relevant primary studies tend to be either cohort or case–control studies, for which the list of potential weaknesses in such observational epidemiological studies, leading to biased estimates of the strength of association either higher or lower than the true value, is rather long and methodologically demanding to apply. Indeed, some of these guidelines list as many as a dozen methodological flaws which should be ruled out by reviewers for each primary study, before assembling a shortlist for potential meta-analytic pooling of results. While such schemata for critically appraising relevant primary studies are helpful in reducing reviewers’ own biases, and improving agreement across SRs by different authors, there remains a substantial amount of *expert judgment* in these assessments of primary study quality. This in turn leads to major discrepancies between various SR shortlists of primary studies suitable for potential pooling of results. Some examples, taken from recently published SR of RF-EMF exposure and various health effects, illustrate major impediments to widespread scientific agreement about causation:

- Some SRs lump biologically quite different health outcomes in one meta-analysis, typically to try to overcome the problem of insufficient numbers of primary studies for any one specific outcome. A clear example is found in two recently published SRs of brain tumors and RF-EMF exposure. Choi et al. (42) and Moon et al. (34) respectively chose to pool, and not to pool studies of pathologically quite different brain tumors—a strategy which would be expected to generally increase across-study heterogeneity in a meta-analysis, thus favoring a decision not to pool. On the other hand, the reviews referenced by Moon and by Choi do not just pool pathologically different cancers, they also pool different exposure metrics—most notably because of different cut-offs in exposure duration and/or cumulative exposure. This makes pooling even less appropriate, as the pooled estimate is uninterpretable.
- Some SRs lump results from cohort and case–control studies, even though these two study designs have substantially different major threats to their internal validity (31, 36, 45). Moon et al. (34) kept these two strata of primary studies separate, but did make use of both strata. Choi et al. (42), on the other hand, chose to exclude from consideration the available cohort studies, on the grounds of serious concerns about the representativeness of the controls, and/or the likely inaccuracy of RF-EMF exposures proxied by the mere recorded possession of a cell phone account [these debates are well covered in the ensuing correspondence with the journal Editors (1, 4, 12) as well as a thorough overview of all these methodological issues (14)]. The approach by Moon et al. (34) of including both case–control and cohort studies is arguably preferable over the Choi et al. (42) approach, of excluding cohort studies, since combining them may “even out” different bias structures in the two study designs.
- Some SRs, and primary studies, explicitly consider the issue of tumor latency, while others do not. This matters because, according to the International Agency for Research on Cancer (56), it is scientifically unjustifiable to rule out cancer causation without having studies spanning at least 30 years since the start of exposure. However, only a handful of studies completed to date have significant statistical power (sample size) over more than a decade or two of follow-up. One pooled analysis of case–control studies covering such a long follow-up period (57) has found that mobile phone use increased the risk of glioma, OR = 1.3 (95% CI = 1.1–1.6 overall) increasing to OR = 3.0 (95% CI = 1.7–5.2) in the >25-year latency group. The OR increased statistically significant both per 100 h of cumulative use, and per year of latency for mobile and cordless phone use.

Therefore, health effects with long latency cannot be confidently established as clearly *not* caused by a given exposure until sufficient time as passed since those exposures appeared in a study population—decades in the case of most solid tumors. For the rapidly changing set of population exposures to RF-EMFS over recent decades, this means in turn that only epidemiological studies of the link between older generations of mobile phone technology (1G through perhaps 2 G and early 3G, but certainly not 4G or 5G) are partially capable of ruling out carcinogenesis, until several more years have passed (7, 10, 19).

It should be noted that both case control and cohort studies are theoretically capable of studying long-latency outcomes, such as tumors without waiting for them to occur (the usual strategy in prospective cohort studies). This can be done by asking at baseline in cohort studies about exposures in the more remote past. However, the accuracy of such self-reported exposures is always less than it is for prospectively recorded exposures, due to “recall bias.” It is also important to note that latencies for any outcome will follow a statistical distribution in time (typically log-normal) so that earlier-occurring tumors, for example, can be expected long before the median latency for that tumor type.

Finally, one important and easily implemented strategy for improving the quality of systematic reviews in this field, and indeed in general, is their preregistration on an appropriate international database of reviews that are planned/in-progress—which helps to reduce the likelihood of undetected publication bias resulting from the failure to publish, especially for studies with largely negative findings (51).

#### Assessing the heterogeneity of primary studies: forest plots

**Figure 1:**
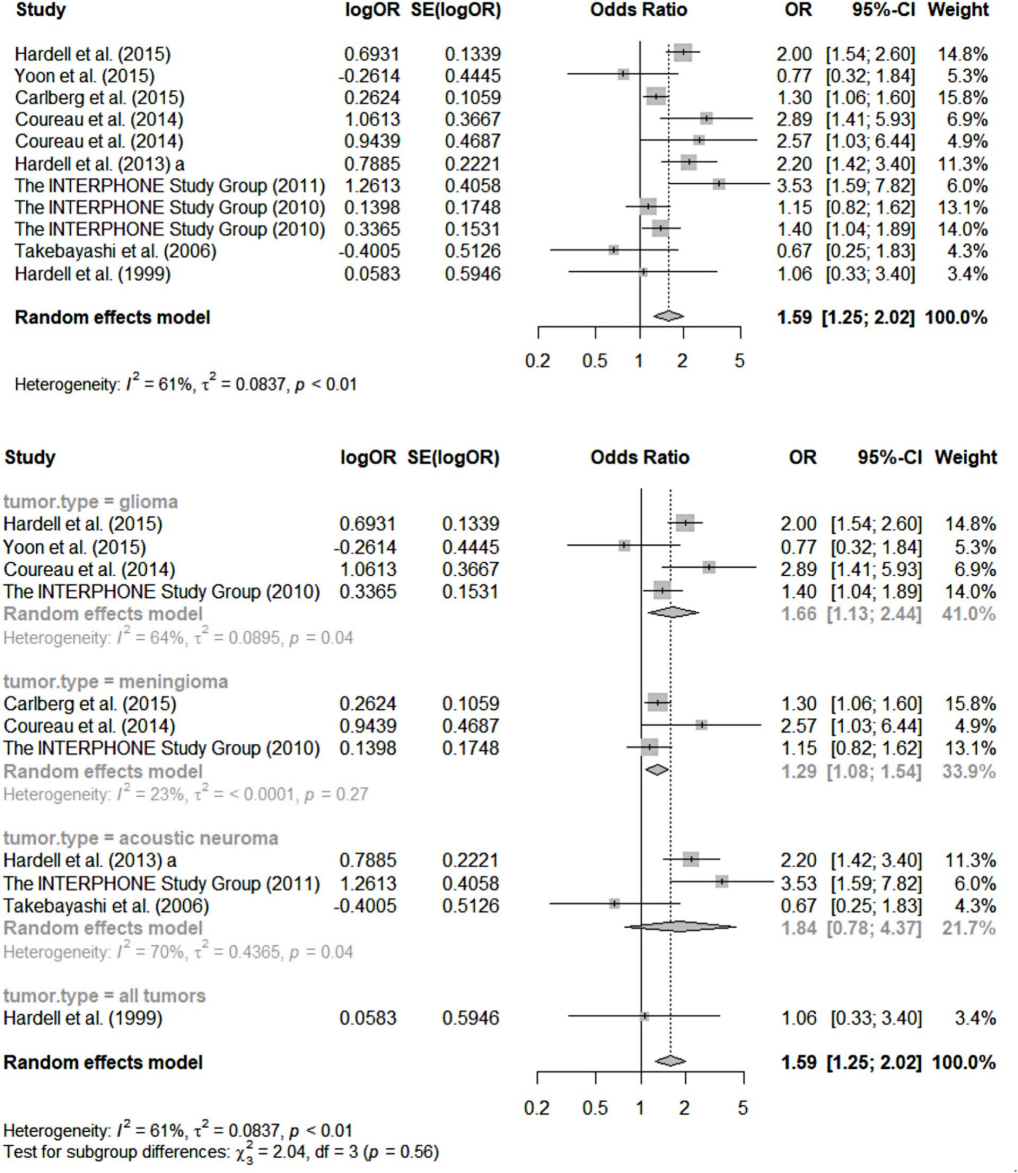
Forest plots for meta-analysis of total cumulative use over 896 h [case–control studies; reprinted with permission from Moon et al., licensed under CC BY 4.0, https://doi.org/10.1186/s12940-024-01117-8; Figure 2 of Moon et al. (34)].

As an illustration of the process used in SRs of assembling only primary studies of reasonable quality and then assessing the consistency of those studies’ methods and findings, a fundamental tool for this purpose, the “forest plot,” is reproduced below (Figure 1) from the SR of Moon et al. (34) focused on RF-EMFs and brain tumors. A well-designed forest plot (such as this one) depicts, across the carefully selected, high-quality primary studies: their main central estimates of the strength of association observed (in this case OR because the Figure includes case–control studies only), on a log scale (plus the log of each OR’s SE); each of those OR estimates’ 95% confidence intervals (both numerically, and as horizontal “whiskers” around the graphed central estimate), quantifying the statistical precision of each RR/OR estimate; the statistical “weight” of each study’s estimate, used in any pooling of the results across the studies (the proportion of any pooled estimate across studies of effect-size contributed by each study, based on its sample-size). Underneath each section of the forest plot is a summary of the meta-analytic statistics for the sub-category of primary studies depicted immediately above, including I-squared and tau-squared heterogeneity statistics (58–60). Finally, on the last line of the graph for each section of the Figure is the pooled estimate of OR and its 95% CI, based on the more conservative “random effects” meta-analytic method of pooling. [For simplicity, since this Figure is intended to merely illustrate and explain the features of a good forest plot, this one only covers one sub-analysis of the case–control studies reviewed by Moon et al. (34), involving these studies’ ORs for total cumulative cell phone use—a metric for total “dose” of RF-EMF exposure, to be further discussed in Section 5—in the highest category analysed: over 896 h.]

For the uninitiated, the following are the key features of this forest plot that not only tell us what this SR/meta-analysis has found, but also reveal quite a bit about those findings’ credibility:

- First and foremost, the number of quality-screened primary studies depicted in Figure 1 for each separate health outcome (type of brain tumor) is relatively small: three for meningioma and acoustic neuroma, and four for glioma. Statistical authorities have recommended that meta-analyses not be conducted (i.e., results across studies pooled) for less than five primary studies at a time (61). The rationale for this recommendation is that the statistical power of standard tests for heterogeneity across primary studies (see next paragraph) is very limited when the number of such studies is less than five; indeed, as Ioannides et al. (62) have pointed out, typical indices of heterogeneity such as I-squared have major uncertainty in their estimates in such circumstances (see second paragraph below).
- Furthermore, the graphical pooling of all 11 studies in the top section of the Figure reveals a relatively high heterogeneity across their OR central estimates, with an I-squared value of 61% (values above 50% are considered suggestive of potentially concerning heterogeneity) so that the wisdom of pooling them at all can be legitimately questioned (58, 60, 63, 64). Such pooling across pathologically quite different tumors surely is clinically and biologically questionable. In essence, such high heterogeneity indicates that these studies were not actually estimating the relative risk linking precisely the same exposure-outcome combination, or at least not doing so using comparable study designs, analyses and approaches to statistical inference. Again, such “substantive” (as opposed to merely “statistical”) across-study heterogeneity is usually considered a contra-indication to pooling of results using meta-analysis. Another recent peer-reviewed SR of this literature did pool all studies of reasonable quality across these same types of brain tumors (42) and has been criticized for it (1, 4), although the original authors have responded vigorously (12).
- The very small number (< five) of studies for each category of tumor, depicted in the bottom half of Figure 1, ha another consequence for those sub-analyses: such small literatures result in I-squared values and other indices of heterogeneity with considerable uncertainty in their estimation—an estimate of which can be calculated but is not provided by Moon et al. (59, 61, 62).
- These weaknesses of the Moon et al. (34) review are compounded by the fact that studies with different high-exposure definitions are also pooled; furthermore, the studies depicted in the forest plot only reflect results for exposures over 896 h—an arguably arbitrary cut-off.
- Overall, there is a clear impression of many studies of insufficient sample size, leading to inadequate statistical precision of the resultant ORs, and contributing to cross-study heterogeneity of findings. While this weakness in a literature can in theory be addressed by—and indeed is the main rationale for—meta-analysis, through the pooling of data across studies, it leads to a kind of instability/fragility of the pooled results, in the sense that just one new, high-quality study of substantial size is quite likely to change the overall result across studies. This translates directly into one of the GRADE criteria for summarizing the confidence one has in the credibility of the meta-analysis, rendering that confidence lower than would otherwise be the case (54).
- Finally, even if we believe the pooled central estimate of OR for, say the 11 studies of diverse brain tumors, From Figure 1 in Moon et al. (34), the result overall OR from such pooling is only 1.59 (95% CI 1.25–2.02)—clearly a “weak effect” which can readily be artefactually created by measurement/information biases, uncontrolled confounding, etc.—hardly a “smoking gun” for causation.
- It may be thought that this unconvincing evidence of causation for RF-EMFs and brain tumors is not the fault of the SR authors, but rather a reflection of the rather immature and sparse literature available (as of 2024) for this review. However, the authors can be faulted for proceeding with a full meta-analysis, which seems contra-indicated, given the above concerns. The authors should have come to the conclusion that a narrative review rather than a pooling of results was the correct approach. In short, “it is too soon to tell” whether causation may be present, rather than clearly the case that it is not present (20, 21, 65). In short, this Figure depicts substantially “iffy” meta-analytic results, which are likely to have led some epidemiologists not to even report the results of pooling the primary studies of separate tumor types.

### Specificity

Most experienced epidemiologists believe that this criterion for causation is rarely met—simply because Nature so rarely matches just one sort of hazardous exposure to a given disease or health outcomes, with just one outcome resulting from that disease. A commonly used example (29, 36) is cigarette smoking: it is linked to dozens of adverse health outcomes of widely varying pathologies, ranging from chronic obstructive pulmonary disease to coronary heart disease, stroke and peripheral vascular disease, as well as several cancers. Yet all of these outcomes have many other known causal exposures, including air pollution in the case of COPD, poor-quality diet, sedentary lifestyle/hypertension/ genetic dyslipidemias in the case of arteriosclerosis outcomes, and a long list of “lifestyle,” environmental and genetic factors in the case of cancers associated with smoking. It is therefore considered a rare epidemiological occurrence when a disease is linked to only one exposure, and *vice-versa*. Indeed, such specificity is commonly found only among infectious diseases (where the causative organism is typically specific to a given clinical picture, although many host-resistance factors also play a causative role), and toxicological syndromes—e.g. the otherwise very rare cancer, mesothelioma, is specific to asbestos exposure (66); however, it has long been known that asbestos exposure also increases the risk of lung cancer and fibrotic lung disease. Often an exposure is wrongly considered to be specific to a rare health outcome only because not enough cohort or case–control studies have been done to investigate other etiological exposures. For example, the industrial pollutant vinyl chloride was long thought to be a specific cause of the rare liver cancer angiosarcoma; however, a more recent review lists several other toxicants as equally implicated and point out that 75% of cases of this rare tumor cannot be linked to any specific toxicant (67). Declaring specificity is therefore inherently fraught, given the impossibility of proving the negative—that no other cause of a disease exists.

There is, however, one particular sense of the term “specific” that is relevant to the literature on the potential causation of adverse health effects, especially neoplasms and RF-EMF exposure: the occurrence of a unique (i.e., virtually pathognomonic) clinical picture obviously anatomically proximal to the suspect source of EMFs. Miller et al. (10) have collected independent reports of extremely localized, multi-focal breast cancers immediately under the position where the patient had carried her cell phone next to the skin for long periods. Because of the well-known inverse square law—which leads to a 100-fold higher EMF exposure intensity at, say, 1 cm distance from breast tissue, compared to, say 10 cm distance when the phone is in a jacket pocket—there would seem to be credibility in inferring that the specificity criterion for causation is met. On the other hand, this phenomenon could also be considered merely a specific instance of “dose response relationship,” another criterion to be further discussed below.

To be fair, there is substantial scientific disagreement about the accuracy with which subjects report the side of their body on which they habitually kept their cell phones over long periods. Karipidis et al. (23, 43) cite studies of brain tumors suggesting that the information biases associated with patients’ reporting of the side of the head on which they held their phone are too major to rely on such analyses. These studies point out that “compensatory” under-reporting of contralateral tumors, found in some analyses, suggests misreporting of the sidedness of phone use by subjects with tumors. This same sort of recall (“rumination”) bias could have affected breast cancer patients “ruminating” over the possible causes of their tumor.

### Temporality

This is by far the easiest criterion to meet, in any causation literature. It merely requires that, as is always the case in cohort studies, and in case–control studies based on historically recorded exposure levels for each subject (as opposed to potentially biased personal recollections), the exposure is measured before the occurrence of the outcome. There are sufficient such studies of RF-EMF exposure and a wide range of health outcomes, including many tumors, to meet this criterion. However, as pointed out above, some potentially important aspects of exposure, such as laterality of usual phone use, can be particularly subject to recall bias in cases within case–control studies.

### Dose–response relationship

Environmental epidemiology has long made much of this criterion for causation, because often it is easier to establish empirically for ambient exposures, especially those with well-defined spatiality. Historically, dose for many environmental hazards tends to have been measured by the proxy “distance” of the subject from the source of the hazards, where this can be accurately determined (68).

However, in the case of RF-EMF exposures, recent case–control and some cohort studies have begun to mathematically estimate the precise exposure to specific body organs, such as the brain, based on a number of assumptions. Karipidis et al. (43, 69) made a major effort to do this, using sophisticated models of EMF-RF exposure from cell phones and state-of-the-art biostatistical methods for formally assessing dose–response relationships (70–72). Published criticism of that approach (21) emphasizes the unclear parameterization of those models and lack of goodness-of -fit statistics in the main WHO SR by Karipidis et al. (43). Karipidis et al. have rebutted those arguments (2024b) in detail. However, the critics have come back with additional concerns (73). The issue is therefore highly contested, and the key evidence around RF-EMF exposure and cancers has now become extremely technical in nature—at least from a policy-maker’s point of view.

An additional challenge to the assessment of primary studies’ handling of dose–response relationships is that virtually all extant primary studies and SRs tend to conflate: (1) true cumulative “dose” of exposure (e.g., as indicated by some measured or modeled strength of the RF-EMF in the relevant body organ /tissue where the tumor under study is located); (2) time-elapsed between exposure and the occurrence of the health outcome under study (i.e., “latency”—see discussion above); and (3) age at first exposure (potentially an important factor in carcinogenesis, as various cancers have different “age-windows” of susceptibility to exposure). Because individuals’ mobile-phone use habits tend to remain rather consistent over many years after acquiring a phone, one would expect study subjects with higher “exposure dosages,” as measured by high cumulative hours of use (e.g., above 1,000 h in the Hardell studies, and the Moon et al. SR) to be virtually the same study subjects as those with the longest duration of use (as usually measured in years), and those with the earliest ages of first exposure. Yet these subjects’ data are critical to the analysis of the latency effects expected for cancers (see above). This greatly complicates the assessment of the adequacy of primary studies’ and systematic reviews’ handling of the three conflated issues. There may be lessons to be learned here from tobacco epidemiology, where the most commonly used measure of “dose” for smoking is “pack-years,” which similarly conflates actual dose (e.g., mean packs per day smoked) and latency. Readers are referred to recent theoretical discussions on these issues (74, 75).

Mention has already been made of some primary studies’ finding of an elevated risk for brain tumors ipsilateral to the side of the head where the cell phone user habitually held his/her phone, compared to the risk for contralateral tumors. The Interphone Study finding, for the highest-exposure-group, of an RR estimate of 3.53—is one of the highest ever observed for any subset of tumors and exposure categories. There are two contrasting views of this finding. As noted above, Karipidis et al. (23, 43) cite studies suggesting that the information biases associated with patients’ reporting of the side on which they held their phone are too major to rely on such analyses. These studies point out that “compensatory” under-reporting of contralateral tumors, found in some analyses, suggests misreporting—recall bias—of the sidedness of phone use by subjects with tumors. Other authors (21) have suggested that such exposure misclassification would generally tend to reduce the observed RR toward the null (RR = 1), making it unlikely that increased RRs would be found purely due to those biases. Certainly, the simplest explanation for an increased ipsilateral risk is the large difference in dose of RF-EMFs emanating from the phone itself, because of the much shorter distance for ipsilateral users between the phone antennae and the side of the brain where the tumor developed, given the inverse square law (7).

In summary then, there is substantial scientific disagreement about whether the extant literature on RF-EMFs and the most frequently studied outcomes—such as brain tumors—demonstrates clear dose–response relationships. One reason for that is no cohort or case–control studies have been able to observe sufficient numbers of subjects for the expected latency period required for many tumors to develop to the point of clinical presentation, which IARC has deemed to be 30 years. The largest numbers of patients followed for the longest follow-up (more than 20 years) are in the COSMOS cohort studies [albeit based on self-reported exposures at baseline recruitment—(76)] and in Hardell’s case–control studies from Sweden [e.g., (57, 77)]. However, the latter relative risk estimates, while elevated to over 3, have relatively wide confidence intervals. This means that more high-quality studies, with longer follow-up, will be needed to resolve both the latency and dose–response issues.

### Biological plausibility

It is under this category of evidence for causation that perhaps the most profound disagreements exist in the scientific community. The history of this disagreement goes back several decades, to the establishment of two contrasting views on what sorts of basic (laboratory- science) biological effects are caused by the levels of RF-EMF exposure widely found in modern society. The one view, held by ICNIRP (the expert body which has advised WHO and many countries on its health and safety “safe exposure” limits for decades) is that there is solid scientific evidence only for heating effects (6, 78–80). The opposite view, held by hundreds of independent scientists, is that there is rapidly mounting evidence of many other biological effects from currently-ambient RF-EMF exposures, including: high levels of oxidative stress leading to cellular damage; changes in cell membrane permeability and function (e.g., through bioelectric effects on ion channels); neuronal dysfunction; and even DNA dysfunction leading to cell dysregulation (3, 8, 10, 19, 81–90). On the other hand, there is rather little evidence that these biological effects actually lead to clear disease outcomes.

One of the challenges of using this Bradford Hill criterion for causation is that it was intended to be integrated with the other, more inherently epidemiological criteria. This challenge has become greater in an era of ever-more-specialized science. For example, Karipidis et al. (24) have pointed out that the pre-determined and published scope of their 2024 SR specifically excluded a detailed review of the laboratory and animal primary studies relevant to RF-EMF exposures and the most commonly studied cancers. A separate review of the laboratory evidence on this question, also commissioned by the WHO, which should appear shortly, given that its protocol was published nearly 3 years ago (91). IARC is said to be planning an update of its 2011 review in the next few years, likely making major use of the WHO SRs. In the interim, it would seem premature for any reasonable scientist to dismiss a causal relationship on the grounds of inadequate evidence for biological plausibility.

### Coherence

This criterion can be most simply stated as “Is the descriptive epidemiological evidence about the spatial and temporal distributions of the exposure, and the outcome, compatible with a causal relationship?” While this may seem a straightforward matter to address empirically in a SR, in practice such a research question is fraught with challenges. A clear example is the relationship between various brain tumors’ incidence and mortality time-trends’ and the presence of preceding changes in RF-EMF exposure prevalence at the population level. Obviously, such data are only available in countries with highly developed systems of national health statistics, especially cancer registries able to consistently tally all cases of all types of tumors reliably over decades, as well as measure the extent of RF-EMF exposure in their entire population at any point in time. Not surprisingly, such countries are few and far between. Some of the most widely cited studies are from Sweden [e.g., (92)]. These appear to show associations between steadily rising rates of cell phone usage in the population, occurring with a credible time-delay (some years) before observed increases in brain tumor incidence, in particular for glioma—perhaps the malignant brain tumor most strongly linked to RF-EMF exposure in epidemiological observational studies.

However, major disagreement has developed on whether such data can be replicated by other investigators in other settings, some with equally well-developed cancer registries. For example, in their WHO-commissioned SR of the more commonly studied cancers, Karipidis et al. (43) analyzed several prior studies on this question and performed sophisticated time-trend simulations to mimic the effects of various reporting biases on observed time trends in brain tumors’ incidence. They concluded that:

“In particular, based on findings from three simulation studies, we could define a credibility benchmark for the observed risk of glioma in relation to long-term mobile phone use, and perform sensitivity meta-analyses excluding studies reporting implausible effect sizes (>1.5) for this exposure contrast.” Karapidis et al. (43), p.43.

In their lengthy critique of this SR by Karipidis et al., Frank et al. (21) stated that overall cancer time trends which combine all cases of similar histology do not capture the unique equipment use and exposure characteristics of the groups in which brain tumor risks were increased in the case–control studies, such as tumor risks in the ipsilateral areas of the brain (temporal lobe) with the highest absorption of RF radiation emitted from a mobile phone held next to the head. It is also a matter of common epidemiological knowledge that specific population subgroups (such as extremely high phone-users, or persons developing very specific subtypes of cancers or cancer locations) may reflect health effects which would not be observable in national time trends data.

Finally, Frank et al. (21) also claimed that key studies finding recent increases in population-level incidence for such brain cancers were omitted by Karipidis et al. (92–96). In their forceful rebuttal to these assertions, Karipidis et al. (23) argued that:

“In some of these studies [cited by Frank et al. (21)] but are actually accompanied by decreases in brain cancers of unspecified site and/or morphology, while overall brain cancer incidence has remained largely unchanged. This suggests improvements in diagnostic techniques as the reason for increasing trends in certain brain cancer sub-types. There have also been shifts in classifying sub-types in updated editions of the WHO classification of tumors of the central nervous system; for example, the WHO 2000 classification induced a shift from anaplastic astrocytoma to glioblastoma. This is addressed in many of the included time-trend simulation studies, e.g., in (21, 113) where reclassification of unclassified or overlapping brain cancers was shown to reduce increased trends in morphological or topological sub-types (such as in glioblastoma multiforme).”

To summarize, as with the state of the evidence cited above for other key Bradford Hill causation criteria, it seems fair to say that there are qualified scientific experts on both sides of the issue of “coherence,” and only highly-technically-trained methodologists are likely to be able to sort out who is right, based on further research.

### Experimental reversibility

Although this criterion for causation is very powerful when met, it is almost impossible to address empirically for disease outcomes generally regarded as “irreversible” once they have been diagnosed—such as cancers. More broadly, one might hope to design a study to show that major reductions in ambient RF-EMF exposures have subsequently led to reductions in related cancers’ incidence. However, no one has yet conducted such a study, largely because no such setting has been identified at the population level, and because of the decades of time-lag required to observe a reduction in cancer incidence after a reduction in exposure, given the long latency involved in carcinogenesis. If we take smoking cessation, for example, as perhaps the best studied reversal of exposure to a proven carcinogen, it is well known from decades-long follow-up of the UK physicians’ cohort of smokers (and quitters) that some common adverse health effects of tobacco show substantially reduced risks within a few months of quitting (e.g., chronic bronchitis) to a few years (e.g., coronary, cerebral and peripheral arteriosclerosis)—whereas the major cancers linked to smoking, such as carcinoma of the lung, persist at elevated incidence rates in quitters, compared to never-smokers, for decades (97).

### Analogy

Finally, this last criterion for causation is deceptively easy to state, but not so easy to fulfill: “Is there an analogous causal relationship established for the exposure in question (i.e., to RF-EMF) and the disease outcome of interest (e.g., brain tumors)—for example in a laboratory animal or credibly similar *in vitro* model?” The most compelling published evidence of such analogies—albeit evidence which has been strongly contested (98, 99)—comes from the results of the USA NIH National Toxicology Program (81, 86, 87) and very similar results of the Ramazzini Institute studies (82, 100) in laboratory rats, showing elevated rates of gliomas and glial cell hyperplasias in the brain and schwannomas and Schwann cell hyperplasias in the heart of exposed male rats [Schwannoma tumors are histologically closely related to acoustic neuromas in humans, linked in other studies to RF-EMF exposure (see previous sections of this paper)]. Additionally, many new studies of potentially adverse short-term RF-EMF effects, related to abnormal physiology/ biochemistry or anatomy observed in the laboratory setting, in either plants or animals, are beginning to appear (3, 8, 10, 83–85, 88–90).

#### More modern methods for assessing causation

Necessarily absent from the original (1965) Bradford Hill criteria for assessing possible causative relationships between exposures and health outcomes are modern epidemiological methods such as Directed Acyclic Graphs (DAGs) (101, 102). Surprisingly, these newer methodological approaches to demonstrating causation appear not to have been utilized in the RF-EMF literature—as yet. Such methods could materially improve the quality of new primary studies, by quantifying the influence of effect moderation and mediation in complex causal chains.

#### The broader issue of potential conflicts of interest

Finally, potential conflicts of interest among authors of the primary studies, and some SRs, has recently become perhaps the most hotly contested issue in this whole field. Peer-reviewed publications by Hardell and Carlberg (7, 83, 84, 103), Hardell and Moskowitz (114), Frank et al. (20, 21), Nordhagen and Flydal (104), and Weller and McCredden (105) have claimed that there is widespread under-declaration of such conflicts of interest, particularly related to research grants and other funding from telecommunications companies with a vested interest in mobile phone and related equipment sales. There are also potential conflicts of interest that are more subtle than merely financially incentivized ones—for example, related to vested interests’ influence on a scientist’s thinking (106). On the other hand, vigorous counterarguments have been published, claiming that there is no hard evidence of potential conflicts of interest among, for example, ICNIRP members or the authors of some of the recently published SRs commissioned by WHO (4, 7, 14, 23–25, 106–108).

More worrisome is the finding of Prasad et al. (109) and Myung et al. (110), that funding source predicted study quality, in their systematic reviews of case–control studies of tumor risk and RF-EMF exposure. Government-funded studies were of higher quality, which in turn was associated with finding a statistically significant association between exposure and brain tumor risk, especially in long-term users (>10 years). Similarly, Carpenter (111) found that empirical studies of RF-EMF exposures and childhood leukemia reported systematically different findings, according to the source of the study’s funding:

“By examining subsequent reports on childhood leukemia it is clear that almost all government or independent studies find either a statistically significant association between magnetic field exposure and childhood leukemia, or an elevated risk of at least OR = 1.5, while almost all industry supported studies fail to find any significant or even suggestive association.” [Abstract]

There is no easy answer to this question. Current guidelines regarding the declaration of potential conflicts of interest are helpful, but there is little “enforcement” and various academic disciplines have varying norms. For example, after decades of published concerns about the undue influence of “Big Pharma” funded researchers in setting public policy, such as the 2014 NICE guidance about who should be prescribed statins (29) medical researchers are quite accustomed to having to declare all potential conflicts of interest in both their publications and prior to participation in influential policy processes. University departments of Engineering and Applied Sciences would appear, on the other hand to regard such funding as not only normal and usual, but in fact a key ingredient in being judged a successful professor. Be that as it may, it would seem only reasonable that all such potential conflicts should be declared at the outset of any scientific interchange.

At a minimum, transdisciplinary fields such as RF-EMF exposures and their biological/health effects will require transdisciplinary agreements about what precisely constitutes potential conflicts of interest. How this might be successfully negotiated is not at all clear.

## Discussion

Based on the application of Bradford Hill’s criteria for causation to the current literature on RF-EMF exposures and adverse health outcomes—especially brain tumors, for which the literature is more voluminous and “mature”—it is clear that many of the primary studies of these associations are problematically afflicted by low-quality science (especially many small studies of low quality), failure of consistent replication and consequent uncertainty about causation. Some epidemiologists (7, 8, 20, 21) have argued that the major methodological weaknesses in this literature, as discussed above, would be expected to produce a bias in the observed strength of association toward the null (i.e., underestimation of the relative risk), particularly the ubiquitous challenge of inaccurate exposure measurement at the individual study subject level, and latency (7, 19, 27, 28, 34). Other epidemiologists have pointed out that the predominant study design in this field—case–control studies—is well known for exaggeration of effect-sizes (relative risks) due to biased recall of exposure in cases, compared to controls, when no objective measurement of exposure is available (23, 43).

Some authors (3, 7, 10, 19) have argued that it is inherently unethical to wait many years for conclusive evidence of causation, before adopting “safe” exposure limits. These researchers invoke the “precautionary principle” which holds that, pending the availability of conclusive science, exposure limits should be set on the basis of *potential* if not necessarily *proven* harms—especially in the case of women of reproductive age and children. Other authorities (7, 79, 80) dispute this view, holding firmly to their long-held conviction that “safe” exposure limits are already in place. There appears to be very little common ground between these views.

### Strengths and weaknesses of this commentary

This commentary—which is not intended to be a narrative let alone systematic review of the pertinent literature on biological and health effects of RF-EMF exposure—has attempted to cover the key issues in that literature related to the assessment of causation, utilizing a wide range of illustrative examples from relevant publications, without any intent or claim to be exhaustive. A strength of this approach is that the detailed discussion above, of the entire nine original Bradford Hill criteria for assessing causation, does provide a relatively neutral, methodologically sound and comprehensive framework for analyzing the critical deficiencies in the current evidence-base in this highly contested field.

### Conclusion and recommendation

Although not a panacea, we propose that a neutral group of international experts in environmental health, nominated by independent scientific and professional bodies, convene a guidelines development process to inform future epidemiological studies, systematic reviews and causal evidence syntheses of associations between RF/EMF and human health outcomes. To overcome entrenched positions identified with various specific experts, an anonymous, Delphi-like consensus -building process might be helpful.

We recognize that several such guidelines are already available, and that the more recent of them [e.g., (49, 50)] are much better suited than previously published guidelines to evaluating observational epidemiological literatures about putative environmental health hazards. However, we believe that further specification of the most common but avoidable “pitfalls” in this field would assist investigators and reviewers to be more vigilant against lower-quality studies which have dominated the field for decades. Wide dissemination of such guidelines could help journals and their reviewers in this field (many of whom appear to be new to epidemiology) to execute, review and publish higher-quality studies, to better inform evidence-based policy. A useful approach for achieving these ends would be to develop a STROBE (Strengthening the Reporting of Observational Studies in Epidemiology) extension specifically for guiding future systematic reviews and primary studies of RF-EMF biological and health effects (55, 112).
